# Supplementary material for: Design, Synthesis and Biological Investigation of Flavone Derivatives as Potential Multi-Receptor Atypical Antipsychotics
Source: Molecules. 2020 Sep 8;25(18):4107. doi: 10.3390/molecules25184107 (PMC7571155; doi:10.3390/molecules25184107)
Supplement: Supplementary file 1 [file molecules-25-04107-s001.pdf]

molecules

MDPI

Supplementary Material

Design, Synthesis and Biological Investigation of Flavone Derivatives as Potential Multi-Receptor Atypical Antipsychotics

Lanchang Gao [1,†], Zhengge Yang [1,†], Jiaying Xiong [1], Chao Hao [1], Ru Ma [2], Xin Liu [1], Bi-Feng Liu [1], Jian Jin [2], Guisen Zhang [1,2,*] and Yin Chen [2,*]

[1] Department of Biomedical Engineering, College of Life Science and Technology, Huazhong University of Science and Technology, Wuhan 430074, China; D201577449@hust.edu.cn (L.G.); charles@long-ou.com (Z.Y.); bear@hust.edu.cn (J.X.); d201880507@hust.edu.cn (C.H.); xliu@mail.hust.edu.cn (X.L.); bfliu@mail.hust.edu.cn (B.-F.L.)

[2] Jiangsu Key Laboratory of Marine Biological Resources and Environment, Jiangsu Key Laboratory of Marine Pharmaceutical Compound Screening, School of Pharmacy, Jiangsu Ocean University, Lianyungang 222005, China; 12111030011@fudan.edu.cn (R.M.); 2019000016@jou.edu.cn (J.J.)

* Correspondence: gszhang@mail.hust.edu.cn (G.Z.); 2019000015@jou.edu.cn (Y.C.); Tel.: +86-27-8779-2235; Fax: +86-27-8779-2170

† These authors contributed equally to this work.

Table of Contents:

1. Receptor Binding Studies **S2– S4**

2. Intrinsic Activity Assessment **S4– S5**

3. hERG Affinity **S5**

4. Acute Toxicity **S5**

5. Behavioral Studies **S6**

6. ^{1}H-NMR, ^{13}C-NMR, HR-MS and HPLC of compound **6j** **S7– S9**

7. References **S9**

1. Receptor Binding Studies

Animals

The experimental animal Chinese Kun Ming (KM) Mice (20 ± 2.0 g) and Sprague-Dawley (SD) rats (250 ± 5.0 g), and all the animals provided with standardized management. Animals were randomly selected and grouped; each group fed separate. All the animals used in the study obey the Ethical Guidelines for the Use of Animals in Research

Materials

The specific radioligands, tissue sources of the test were used as following:D_2 receptor: rat striatum, ^{3}H-spiperone; D_3 receptor: rat olfactory tubercle,^{3}H-7-OH-DPAT; 5-HT_{1A} receptor: rat brain cortex,^{3}H-8-OH-DPAT; 5-HT_{2A} receptor: rat brain cortex, ^{3}H-ketanserin; 5-HT_{2C} receptor: rat brain cortex, ^{3}H-mesulergine; α_1 receptor: rat cerebral cortex,^{3}H-prazosin; H_1 receptor: guinea pig cerebellum, ^{3}H-mepyramine. All the radioligands were bought from Sigma Co., Ltd. (Knoxville, Austrialia). Tris, NaCl, KCl, $MgCl_2$, $CaCl_2$, potassium dihydrogen phosphate, sodium hydroxide, ascorbic acid, pargyline and other chemical reagents were of analytical grade and obtained from Sinopharm Chemical Reagent Co., Ltd. (Shanghai, China)

Instrumentation and Consumables

pH meter (PHS-3C, Shanghai, China), precision electronic balance (MS105DU, Mettler Toledo, Columbus, OH, USA), Electrically-driven tissue homogenizer (ULTRA TURAX T18 basic, IKA, Staufen, Germany), vortex mixers (XH-C, Shanghai, China), large-capacity high-speed refrigerated centrifuge (SIGMA 3K15, Neustadt, Germany), scintillation counter (LS 6500, Beckman, Brea, CA, USA), electro-heating standing-temperature cultivator, −80 °C refrigerator (Haier, Qingdao, China), commercial ice machine, scintillation solution (MICROSCINT PS, PerkinElmer, Waltham, MA, USA), Unifilter-96 GF/B (Perkin Elmer), fiberglass filter paper (GF/C Whatman, Maidstone, United Kingdom), patch clamp amplifier (Axopatch 200B, Molecular Devices, Axon, Scottsdale, AZ, USA), digital converter (Digidata 1440A, Molecular Devices), recording electrodes (BF120-94-15, Sutter Instrument Company, Novato, CA, USA), pCLAMP software (version 10.1, Molecular Devices).

General Procedures for the Binding Assays

All of the new compounds were dissolved in 50% (v/v) DMSO and the compound concentration was adjusted to 2×10^{-3} M; dilution to the initial concentration of the new compound, 2×10^{-4} M, contained 5% DMSO. Each specific binding (SB) was calculated as the total binding (TB) minus the nonspecific binding (NB) at a particular concentration of radioligand. Each percentage of inhibition (%) was calculated as follows: percentage of inhibition (%) = [(TB – CB)/(TB – NB)] × 100.

Blank binding experiments contained 0.25% (v/v) DMSO were performed; DMSO had no effect. All compounds were tested at least three times over a 6-fold concentration range (10^{-5} M to 10^{-10} M). IC_{50} values were determined by nonlinear regression analysis with fitting to the Hill equation curve. K_i values were calculated using the Cheng and Prussoff equation, $K_i = IC_{50}/(1 + C/K_d)$, where C represents the concentration of the hot ligand used and K_d the receptor dissociation constant of each labeled ligand. The mean K_i values and SEM were derived in at least three independent experiments.

D_2 Receptor [1-3].

The rats were cut off the head in ice, take out the cerebral cortex and place in centrifuge tubes, and then homogenized in 20 volumes of ice-cold Trise HCl buffer (50 mM, pH 7.5) using an ULTRA TURAX homogenizer, then centrifuged at 48000 *g* for 10 min at 4 °C, this procedure were repeated twice and the resulting precipitate was preserved and the supernatant discarded. The final precipitate was resuspended in 50 mM ice-cold Tris-HCl containing 120 mM NaCl, 5 mM KCl, 2 mM $CaCl_2$, 1 mM $MgCl_2$, 0.1% ascorbic acid, and 5 mM pargyline. For total binding, to each assay tube

was added 900 μL of the tissue suspension, 50 μL of 0.5 nM^{3}H-spiperone (16.2 Ci/ mmol; PerkinElmer Life Sciences, Boston, MA, USA) and 50 μL Tris HCl buffer containing 120 mM NaCl, 5 mM KCl, 2 mM $CaCl_2$, 1 mM $MgCl_2$, 0.1% ascorbic acid, and 5 mM pargyline. For nonspecific binding, to each assay tube was added 900 μL of the tissue suspension, 50 μL of ^{3}H-spiperone, and 50 μL of 10 mM (*t*)-butaclamol. For compound binding, to each assay tube was added 900 μL of the tissue suspension, 50 μL of ^{3}H-spiperone, and 50 μL of new compounds or reference drug. The tubes were incubated at 37 °C for 30 min. The tubes were incubated at 37 °C for 30 min. the incubation filtered through Whatman GF/B glass filters, filtrates were washed twice with 5.0 mL cold buffer solution, then transferred to a 3.0 mL scintillation vials, 1.0 mL scintillation solution added and the mixture was detected by Beckman LS 6500 liquid scintillation counter.

D_3 Receptor [1-3].

The rats' heads were removed in ice, the rat olfactory system removed and placed in centrifuge tubes, and then homogenized in 20 volumes of ice-cold Tris HCl buffer (50 mM, pH 7.5) using an ULTRA TURAX homogenizer, followed by centrifugation at 48000 *g* for 10 min at 4 °C, this procedure were repeated twice and the resulting precipitate was preserved and supernatant discarded. The final precipitate was resuspended in 50 mM Hepes Na, pH 7.5, containing 1 mM EDTA, 0.005% ascorbic acid, 0.1% albumin, and 200 nM eliprodil. For total binding, to each assay tube was added 900 μL of membranes, 50 μL of 0.6 nM^{3}H-7-OH-DPAT (50 Ci/mmol; PerkinElmer Life Sciences), and 50 μL of 50 mM Hepes Na, pH 7.5, containing 1 mM EDTA, 0.005% ascorbic acid, 0.1% albumin, and 200 nM eliprodil. For nonspecific binding, to each assay tube was added 900 μL of membranes, 50 μL of ^{3}H-7-OH-DPAT (50 Ci/mmol; PerkinElmer Life Sciences), and 50 μL of 1 μM dopamine. For specific binding, to each assay tube was added 900 μL of membranes, 50 μL of ^{3}H-7-OH-DPAT (50 Ci/mmol; PerkinElmer Life Sciences), and 50 μL of new compounds or reference drug. The tubes were incubated at 25 °C for 60 min. The subsequent testing operation as described for the bending of dopamine D_2 receptor.

5-HT_{1A} Receptor [1-3].

The rats' heads were cut off in ice, the cerebral cortex removed and placed in centrifuge tubes, and then homogenized in 20 volumes of ice-cold Tris HCl buffer (50 mM, pH 7.5) using an ULTRA TURAX homogenizer, followed by centrifugation at 32000 *g* for 10 min at 4 °C, the resulting precipitate was preserved and supernatant discarded. The precipitate was then resuspended in the Trise HCl buffer again, incubated for 10 min at 37 °C, and centrifuged at 32000 g for 10 min. The precipitate was resuspended in Tris HCl buffer containing 10 mM Pargyline, 4 mM $CaCl_2$ and 0.1% ascorbic acid. Total binding each assay tube was added 900 mL of the tissue suspension, 50 mL of 0.5 nM^{3}H-8-OH-DPAT (187.4 Ci/mmol, Perkin Elmer Life Sciences), 50 mL Tris HCl buffer containing 10 mM pargyline, 4 mM $CaCl_2$ and 0.1% ascorbic acid. Non-specific binding each assay tube was added 900 mL of the tissue suspension, 50 mL of ^{3}H-8-OH-DPAT, 50 mL of 10 mM serotonin. Specific binding each assay tube was added 900 mL of the tissue suspension, 50 mL of ^{3}H-8-OH-DPAT, 50 mL of new compounds or reference drug. The tubes were incubated at 37 °C for 30 min. The subsequent testing operation as described for the bending of dopamine D_2 receptor.

5-HT_{2A} Receptor [1-3].

The rats' heads were cut off in ice, the cerebral cortex removed and placed in centrifuge tubes, and then homogenized in 20 volumes of ice-cold Tris HCl buffer (50 mM, pH 7.5) using an ULTRA TURAX homogenizer, followed by centrifugation at 32000 *g* for 20 min at 4 °C, the resulting precipitate was preserved and supernatant discarded. The precipitate was then resuspended in the Trise HCl buffer again, incubated for 10 min at 37 °C, and centrifuged at 32000 *g* for 20 min. The final precipitate was resuspended in 50 volumes of the Tris HCl buffer. Total binding each assay tube was added 900 mL of the tissue suspension, 50 mL of 0.6 nM^{3}H-ketanserin (60.0 Ci/mmol, Perkin Elmer Life Sciences), 50 mL Trise HCl buffer. Non-specific binding each assay tube was added 900 mL of

the tissue suspension, 50 mL of ^{3}H-ketanserin, 50 mL of 10 mM methisergide. Specific binding each assay tube was added 900 mL of the tissue suspension, 50 mL of ^{3}H-ketanserin, 150 mL of new compounds or reference drug. The tubes were incubated at 37 °C for 30 min. The subsequent testing operation as described for the bending of dopamine D_2 receptor.

5-HT$_{2C}$ Receptor [1-3].

The rats' heads were cut off in ice, the cerebral cortex removed and placed in centrifuge tubes, and then homogenized in 20 volumes of ice-cold Tris HCl buffer (50 mM, pH 7.5) using an ULTRA TURAX homogenizer, followed by centrifugation at 32000 g for 20 min at 4 °C, the resulting precipitate was preserved and supernatant discarded. The precipitate was then resuspended in 50 vol of Tris HCl buffer. For total binding, to each assay tube was added 900 μL of the tissue suspension, 50 μL of ^{3}H-mesulergine, 50 mL of spiperone, and 50 μL of Tris-HCl buffer. For nonspecific binding, to each assay tube was added 900 μL of the tissue suspension, 50 μL of 1 nM ^{3}H-mesulergine (85.4 Ci/mmol; PerkinElmer Life Sciences), 50 μL of spiperone, and 50 μL of 10 mM mianserin. For compound binding, to each assay tube was added 900 μL of the tissue suspension, 50 μL of ^{3}H-mesulergine, 50 μL of spiperone, and 50 μL of new compounds or reference drug. The tubes were incubated at 37 °C for 15 min. The subsequent testing operation as described for the bending of dopamine D_2 receptor.

A$_1$ Receptor [1-3].

The rats' heads were cut off in ice, the cerebral cortex removed and placed in centrifuge tubes, and then homogenized in 20 volumes of ice-cold Tris HCl buffer (50 mM, pH 7.5) using an ULTRA TURAX homogenizer, followed by centrifugation at 44000 *g* for 20 min at 4 °C, the resulting precipitate was preserved and supernatant discarded. The precipitate was then resuspended in 50 vol of Tris HCl buffer. For total binding, to each assay tube was added 900 μL of the tissue suspension, 50 μL of 1nM^{3}H-prazosin (85.4 Ci/mmol; PerkinElmer Life Sciences), and 50 μL of Tris-HCl buffer. For nonspecific binding, to each assay tube was added 900 μL of the tissue suspension, 50 μL of 1 nM ^{3}H-prazosin, and 50 μL of 10 mM prazosin. For compound binding, to each assay tube was added 900 μL of the tissue suspension, 50 μL of ^{3}H-prazosin, and 50 μL of new compounds or reference drug. The tubes were incubated at 25 °C for 60 min. The subsequent testing operation as described for the bending of dopamine D_2 receptor.

H$_1$ Receptor [1-3].

The rats' heads were cut off in ice, the cerebral cortex removed and placed in centrifuge tubes, and then homogenized in 20 volumes of ice-cold Tris HCl buffer (50 mM, pH=7.5) using an ULTRA TURAX homogenizer, following centrifuged at 44000 *g* for 20 min at 4 °C, this procedure were repeated twice and the resulting precipitate was preserved and supernatant discarded. The final precipitate was then resuspended in 50 vol of phosphate buffer. Total binding each assay tube was added 900 mL of membranes 50 mL of 1 nM^{3}H-mepyramine (20.0 Ci/mmol; Perkin Elmer Life Sciences), 50 mL phosphate buffer. Non-specific binding each assay tube was added 900 mL of membranes, 50 mL of ^{3}H-mepyramine, 50 mL of 1 mM promethazine. Specific binding each assay tube was added 900 mL of membranes, 50 mL of ^{3}H-mepyramine, 50 mL of new compounds or reference drug. The tubes were incubated at 30 C for 60 min. The subsequent testing operation as described for the bending of dopamine D_2 receptor.

2. Intrinsic Activity Assessment [3].

HEK cells expressing five receptors (HEK293/ D_{2L}, HEK293/ACTOne D_3, HEK293/5-HT$_{1A}$, HEK293/5-HT$_{2A}$, and HEK293T/h5-HT6) were seeded in a 384-well black-walled, clear bottom plate at a density of 1.5×10^4 cells/well in cell seeding medium (90% DMEM and 10% dialyzed serum) and incubated in CO_2 incubator for 16-24 h. For the D_{2L} assay, all compounds were diluted with DMSO, 1/2 log dilution (3.17-fold), 11 points and triplicate to get the compounds dose, then the assay buffer

added to get the working concentration and do the test. Agonist mode: (1) Diluted the reference compound dopamine to 50 μM (11 points, 5×), (2) diluted the test compounds to working concentration (11 points, 5×). Antagonist mode: (1) Diluted the reference compound SCH23390 to 600 μM (11 points, 6×), (2) diluted the test compounds to working concentration (11 points, 6×). Assay buffer: 1× HBSS, 20 mM HEPES, 2.5 mM probenecid (probenecid is 400 mM stock in 1 M NaOH, add freshly). Assay buffer was used as dye loading buffer, compound dilution buffer, etc., and then gently discarded the medium, and 20 μL of calcium dye loading solution was added into each well. The plate at 37 °C and incubated in the dark for 60 min before calcium signal read out.

For the agonist assay, 5 μL/well 5× working concentration of test compounds was added into cell plate using FLIPR and read with FLIPR (FLIPR Calcium 4, Molecular Devices) using the specified settings and saved data. The total assay volume was 25 μL including 20 μL/well dye loading buffer and 5 μL/well 5× working concentration of test compounds. For the antagonist assay, 6× working concentration of antagonist compound at 5 μL/well was added to cells and incubated plate at room temperature in the dark for 15 min, transferred to the assay plate to FLIPR, and 5 μL/well 6× working concentration of antagonist compound added was using FLIPR and read with FLIPR using the specified settings and saved data. The total assay volume was 30 μL including 20 μL/well dye loading buffer, 5 μL/well 6× work concentration of test compounds, and 5 μL/well 6× work concentration of agonist compound. Data analysis: FLIPR read the plate and got the maximal fluorescence signal data from the excitation light wavelength at 480 nm and emission light wavelength at 520 nm. All results for test compounds were test three times. According to the positive control (HPE) and negative control (ZPE) results, the effect (%) or inhibition (%) of reference and the test compounds was calculated by using Graph Pad Prism 5 to analyze the data and obtained the dose response curve and the value of EC_{50} and IC_{50}. Effect (%) for agonist mode was calculated from the following equation:

$$\text{effect (\%)} = (\text{value}_{\text{(raw data)}} - \text{average}_{\text{(ZPE)}}) / (\text{value}_{\text{(HEP)}} - \text{average}_{\text{(ZPE)}}) \times 100$$

The % effect was then plotted as a function of the log of the cumulative doses of compounds. Inhibition (%) for antagonist mode was calculated from the following equation:

$$\text{inhibition (\%)} = (\text{average}_{\text{(HEP)}} - \text{average}_{\text{(raw data)}}) / (\text{value}_{\text{(HEP)}} - \text{average}_{\text{(ZPE)}}) \times 100$$

The % inhibition was then plotted as a function of the log of the cumulative doses of compounds.

3. hERG Affinity [2,3]

The ability to block hERG potassium channels was determined according to the related literatures our previous reported [1-3]. The test compound **6j** was dissolved in 50% (w/v) DMSO and the initial concentration was 1 mM, and the test concentration for compound **6j** was diluted to different drug concentrations (0.3, 1, 3, and 10 μM) by the bath solution. The holding membrane(HEK cells) potential was switched from −80 to +50 mV for 2 s, followed by return to −50 mV for 3 s (sampling rate of 4 kHz, low-pass filtered at 1 kHz) in intervals of 30 s. Tail currents were measured at −50 mV in control and in the presence of the drug at different concentrations determined empirically. All raw measurements were performed using Clamp fit (version 10.2), a part of pCLAMP software (version 10.1). The hERG inhibition experiments were tested for three independent experiments. Results were transferred to the program Statistical Package for the Social Sciences (SPSS) spreadsheets for further analysis.

4. Acute Toxicity

The mice were randomly divided into several groups(n=10), each group were treated with increasing doses of the compound **6j** (250, 500, 1000, 1500, and 2000 mg/kg). The number of surviving animals was recorded until 24 h after giving **6j**, and the relative mortality rates of each group was calculated. The LD_{50} values were calculated by using the Statistical Package for the Social Sciences (SPSS) program (SPSS Inc., Chicago, IL, USA).

5. Behavioral Studies

5.1. Apomorphine-Induced Climbing [1-3].

The mice were randomly divided into several groups (n=10), and treated with increasing doses of the clozapine (1.0, 3.0, 10.0 and 30.0 mg/kg), risperidone (0.01, 0.03, 0.1, and 0.3 mg/kg), and compound **6j** (0.01, 0.03, 0.1, and 0.3 mg/kg), respectively. After an hour, the mice given subcutaneous injection with 1.0 mg/kg of the apomorphine in 0.9% NaCl + 0.1% ascorbic acid, placed in cylindrical wire cages (12 cm in diameter, 14 cm in height), and observed for climbing behavior at 10, 20, and 30 min post dose. The climbing behavior was scored as follows: 3 or 4 paws on the cage floor = 0 score; 2 and 3 paws on the cage = 1 score; 4 paws on the cage = 2 score. The statistical significances of drug effects were analyzed by the nonparametric two-tailed-Mann-Whitney U test: #, $p < 0.05$ versus vehicle treatment; **, $p < 0.01$, *, $p < 0.05$ versus apomorphine treatment.

5.2. MK-801-Induced Hyperactivity [1-3].

The mice were randomly divided into several groups(n=10), and treated with increasing doses of the clozapine (0.3, 1.0, 3.0 and 10.0 mg/kg), risperidone (0.005, 0.015, 0.045 and 0.135 mg/kg), and compound **6j** (0.05, 0.15, 0.45 and 1.35 mg/kg), respectively. Animals were placed in Plexiglas cages for evaluating locomotor activity. After an hour, the mice given subcutaneous injection with 0.3 mg/kg of MK-801 and the locomotor activity of each animal was recorded for 90 min. Statistical evaluation was performed by Two Way ANOVA followed by Tukey test for multiple comparisons. #, $p <0.05$ versus vehicle treatment; **, $p < 0.01$, *, $p < 0.05$ versus MK-801treatment.

5.3 Catalepsy Test [1-3].

The mice were randomly divided into several groups(n=10), and treated with increasing doses of the clozapine (1.0, 5.0, 15.0, 45.0 and 100.0 mg/kg), risperidone (0.1, 0.3, 1.0 and 3.0 mg/ kg), and compound **6j** (1.0, 5.0, 15.0, 45.0 and 100.0 mg/kg), respectively. Catalepsy was evaluated on a metal bar 0.3 cm in diameter positioned 4.5 cm above the tabletop. The test consisted in positioning the animal with its forepaws on the bar and recording how long it remained hanging onto the bar; the end point was 60 s and an all-or-none criterion was used. A mean immobility score of 30 s was used as the criterion for the presence of catalepsy.

6. ^{1}H-NMR of Compound 6j

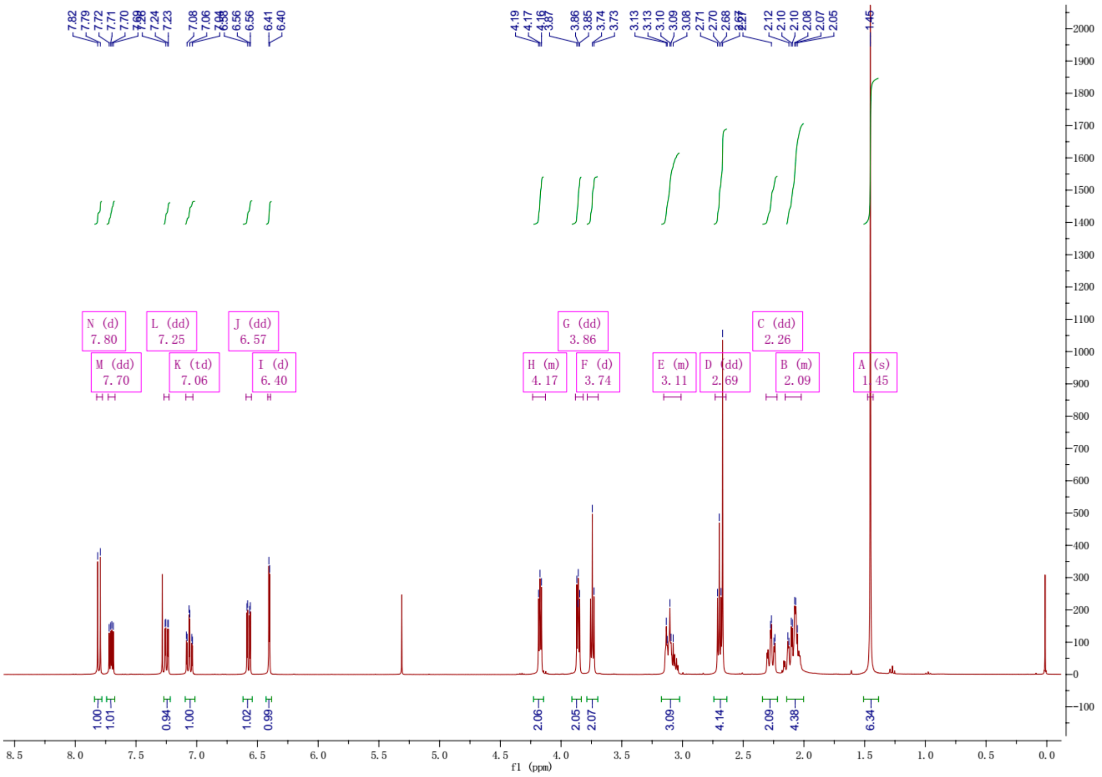

^{13}C-NMR of Compound 6j

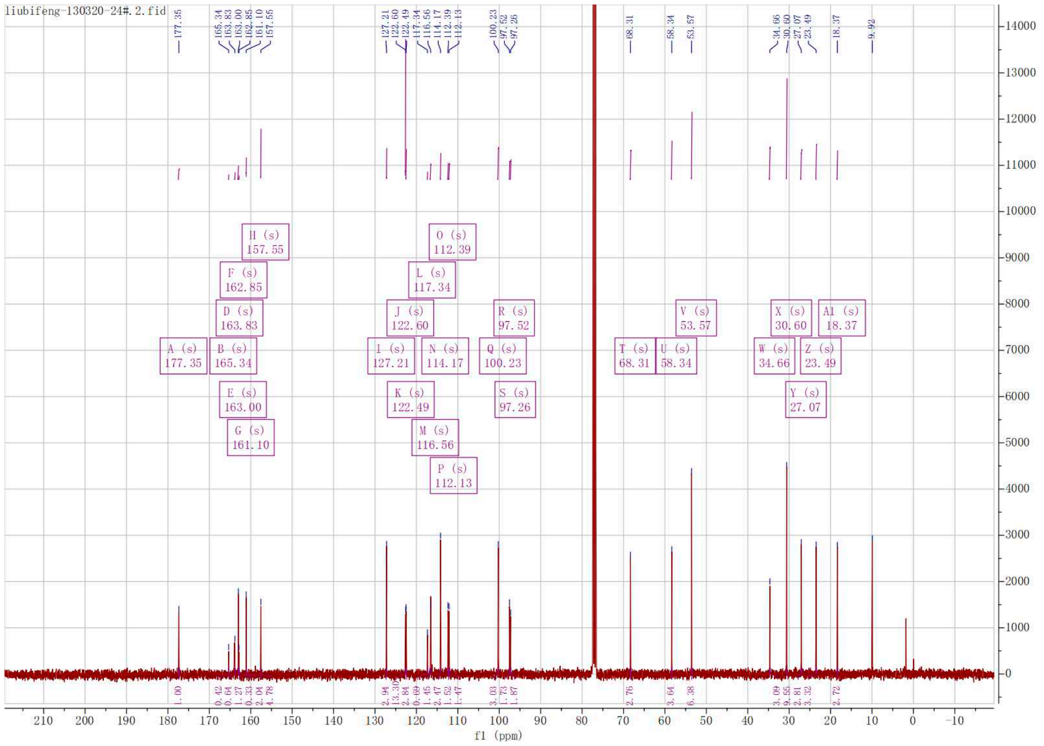

HR-MS of the compound 6j

User Spectra

Fragmentor Voltage	Collision Energy	Ionization Mode
80	0	ESI

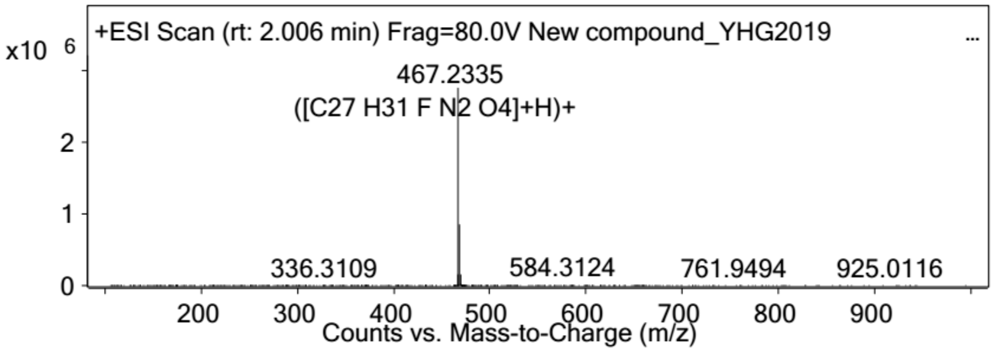

Peak List

m/z	z	Abund	Formula	Ion
467.2335	1	2767933.25	C27 H31 F N2 O4	(M+H)+
467.4083		149728.33		
468.2368	1	848469.69	C27 H31 F N2 O4	(M+H)+
469.2405	1	149865.5	C27 H31 F N2 O4	(M+H)+
470.2426	1	16837.03		
489.2146	1	8419		
490.2166	1	2666.26		
505.1894	1	3669.95		
526.4312	1	4123.55		
584.3124	1	5379.24		

Formula Calculator Element Limits

Element	Min	Max
C	20	30
H	20	35
O	0	5
N	2	2
S	0	0
Cl	0	0
P	0	0
F	0	1
I	0	0

Formula Calculator Results

Formula	Best	Mass	Tgt Mass	Diff (ppm)	Ion Species	Score
C27 H31 F N2 O4	True	466.2263	466.2268	1.11	C27 H32 F N2 O4	99.15
C30 H30 N2 O3	False	466.2263	466.2256	-1.32	C30 H31 N2 O3	97.86

--- End Of Report ---

7. References

1. Chen, Y.; Wang, S.; Xu, X.; Liu, X.; Yu, M.; Zhao, S.; Liu, S.; Qiu, Y.; Zhang, T.; Liu, B. F.; Zhang, G. Synthesis and biological investigation of coumarin piperazine(piperidine) derivatives as potential multireceptor atypical antipsychotics. *J. Med. Chem*. **2013**, *56*, 4671-4690.
2. Chen, Y.; Lan, Y.; Wang, S.; Zhang, H.; Xu, X.; Liu, X.; Yu, M.; Liu, B. F.; Zhang, G. Synthesis and evaluation of new coumarin derivatives as potential atypical antipsychotics. *Eur. J. Med. Chem.* **2014**, *74*, 427–439.
3. Cao, X,; Zhang, Y.; Chen, Y.; Oiu, Y.; Yu, M,; Xu, X,; Liu, X,; Liu, B. F.; Zhang, G. Synthesis and biological evaluation of fused tricyclic heterocycle piperazine (piperidine) derivatives as potential multireceptor atypical antipsychotics. *J. Med. Chem.***2018**, *61*, 10017-10039.

© 2020 by the authors. Licensee MDPI, Basel, Switzerland. This article is an open access article distributed under the terms and conditions of the Creative Commons Attribution (CC BY) license (http://creativecommons.org/licenses/by/4.0/).
